# Risk stratification by donor-derived cell-free DNA: a three-year single center study of kidney transplant outcomes from 257 patients

**DOI:** 10.3389/fimmu.2026.1737024

**Published:** 2026-02-13

**Authors:** Rhys Mendel, Kabir Jalal, Lin Liu, Ying Huang, Cynthia Wan, Eric Zou, Olivia Sorci, Kanishka Mohib, Samer Kareem, John E. Tomaszewski, Jon R. Von Visger, Richard Quigg, Shirley Chang, Xiaoyan Wu

**Affiliations:** 1 Jacobs School of Medicine and Biomedical Sciences, University at Buffalo, Buffalo, NY, United States; 2 Department of Biostatistics, University at Buffalo, Buffalo, NY, United States; 3 Department of Pathology and Anatomical Sciences, Jacobs School of Medicine & Biomedical Sciences, Buffalo, NY, United States; 4 University of Virginia School of Medicine, Charlottesville, VA, United States; 5 North Carolina School of Sciences and Mathematics, Durham, NC, United States; 6 Department of Internal Medicine, University of Rochester School of Medicine & Dentistry, Rochester, NY, United States; 7 Research and Development, CareDx Inc., Brisbane, CA, United States; 8 Department of Medicine, Jacobs School of Medicine & Biomedical Sciences, Erie County Medical Center, Buffalo, NY, United States; 9 Department of Pediatric Nephrology, Jacobs School of Medicine and Biomedical Sciences, Oishei Children’s Hospital, Buffalo, NY, United States

**Keywords:** acute antibody mediated rejection, acute cellular rejection, allograft rejection, Dd-cfDNA, kidney transplant, multiple variant analysis, non-invasive diagnosis, survival analysis

## Abstract

**Background:**

Donor-derived cell-free DNA is an established blood-based biomarker used to assess alloimmune activity after kidney transplantation.

**Methods:**

We performed a retrospective study of 257 kidney transplant recipients that had at least one dd-cfDNA measurements during 3-year period. The primary aim was to assess the association between dd-cfDNA levels with graft function and survival. Secondary exploratory aims included examining the relationships between dd-cfDNA strata and biopsy-proven rejection, donor-specific antibodies, C4d deposition, and longitudinal eGFR trajectories. Patients were stratified by their highest dd-cfDNA measurement into three groups: <0.50%, 0.50–0.99%, and ≥1.0%.

**Results:**

Patients categorized in the ≥1.0% dd-cfDNA group had increased rates of rejection, more severe histopathologic injury, and a higher prevalence of DSAs. With the ≥ 0.50-0.90% dd-cfDNA group having greater variability and decline in eGFR over the 3 year period. In exploratory multivariable modeling, higher dd-cfDNA strata were associated with a decline in graft function and survival.

**Conclusions:**

Elevated dd-cfDNA levels were associated with adverse alloimmune and functional outcomes, including rejection, DSA positivity, and reduced graft survival (p=0.0071). A logistic regression model identified eGFR decline in the >1.0% group to predict long-term graft failure and patient survival (p=0.04). These findings support the clinical value of dd-cfDNA as a biomarker of alloimmune risk in kidney transplant recipients.

## Introduction

Kidney transplantation remains the preferred intervention for individuals diagnosed with or nearing end-stage kidney disease (ESKD). In 2022 alone, a total of 25, 498 kidney transplants were performed in the United States – a 3.4 percent increase over 2021 (United Network for Organ Sharing, 2022). Kidney transplantation is associated with reduced morbidity and mortality, as well as an improved quality of life compared to individuals undergoing long-term dialysis therapy (1, 2).

Advancements in kidney transplantation, such as immunosuppressive therapy, surgical techniques, and post-transplant care are routine procedure (3). Despite this progress, allograft rejection remains a significant challenge. Approximately 10% of living donor and 20% of deceased donor kidney transplants do not reach the five-year survival mark post-transplant, with kidney transplant rejection being a substantial factor contributing to allograft dysfunction and potential loss (4).

Subclinical acute rejection (subAR) is a significant challenge in kidney transplantation. SubAR includes subclinical T-cell mediated rejection (SC-TCMR) and antibody-mediated rejection (SC-AMBR). Studies show that in the first and second years following a kidney transplant, subAR was detected in up to 25% of protocol biopsies and 35% of patients, respectively (5–7). As of 2021, less than half of major transplant centers across the United States perform protocol biopsies (8). These biopsies, conducted at clinically predefined intervals in stable patients, aim to detect subAR. However, reluctance to adopt surveillance biopsies may partly be due to a lack of definitive studies showing a clear benefit to improving long-term outcomes (9). Indeed, a meta-analysis examining several randomized control trials, showed that there is no benefit to detecting acute rejection or improving outcomes at 12 months when surveillance biopsies are performed (9). Therefore, their utilization is less frequent than indication biopsies, which assess patients experiencing an acute or chronic decline in renal function, often prompted by laboratory results such as increased serum creatinine or *de novo* donor specific antibody (dnDSA).

The challenges associated with both protocol and indication biopsies, including burdens on patients and the risk of missing subAR, can lead to delayed treatment. Instead, established standard of care diagnostic tools are used to monitor patients post-transplant including serum creatinine, donor specific antibodies (DSA), and proteinuria. However, commonly used biomarkers have major limitations and do not represent a direct measure as to the level of injury that the kidney is undergoing. For example, serum creatinine lacks sensitivity and specificity in identifying allograft injury and can be affected by factors such as diet, nutritional status, muscle mass, and sex (10–12). Moreover, serum creatinine often is a lagging indicator for changes in glomerular filtration rate until a steady state has been reached (13–15). Generally, more than 50% of kidney function must be lost before serum creatinine begins to rise, which could take weeks to months (13, 14). The presence of donor-specific antibodies can be non-specific and lacks a direct association with the severity of rejection or even presence of rejection (16, 17). On the other hand, proteinuria has been shown to be lagging indicators of kidney injury and usually associated with the significant functional loss of the kidney (18, 19). Therefore, the use of non-invasive biomarkers that can quantitatively reflect kidney injury, subAR or fulminant rejection could be crucial to timely intervention and prolongation of graft function and survival.

Donor-derived cell-free DNA (dd-cfDNA) has emerged as a promising non-invasive screening tool to detect injury incurred to the allograft and used to identify rejection (20). AlloSure, the first clinically validated dd-cfDNA test, was introduced in 2016 (20, 21). It quantitatively measures dd-cfDNA in the blood (serum) of transplant patients using next-generation sequencing (NGS) of pre-defined single-nucleotide polymorphisms (SNPs) (21). The DART study established that dd-cfDNA is released at a low level in stable patients (<0.5%) and is elevated >1% in patients with biopsy proven rejection (20). Since the DART study, numerous publications have demonstrated the value of dd-cfDNA in helping manage kidney transplant patients and have confirmed those findings (22–24). The usefulness of dd-cfDNA was initially based on a very high negative predictive value to avoid unnecessary biopsies triggered by standard clinical diagnostics tests in the for-cause setting. However, the ADMIRAL study showed the value of use of dd-cfDNA the in-surveillance setting demonstrating that an increase of 149% from baseline levels are indicative of clinically significant injury (22). By establishing a baseline level for individual patients and examining the relative change over time it was possible to detect subAR (25–27). Using a baseline dd-cfDNA and monitoring relative change in levels over time resulted in early detection of rejection compared to any other biomarker. dd-cfDNA elevations over time were shown to precede antibody mediated rejection, cellular rejection or rise of dnDSA, in several different types of solid organ recipients (heart, lung kidney), ranging from months to weeks prior to detection by the index biopsy or other standard of care diagnostic tests (22, 23, 28, 29).

The use of dd-cfDNA also expanded to managing patients requiring immune modulation either due to viral infections or malignancy (30, 31). More recently, Aubert et al. performed a large multi-national - multi-center study which established dd-cfDNA as an independent factor for identifying rejection in kidney transplant recipients in various clinical scenarios and settings (27). Additionally, they found incrementally higher levels of dd-cfDNA correlate with higher degree of tissue injury as noted by histopathology grades (27). Overall, the study showed that combining dd-cfDNA with standard of care parameters can results in a significant improvement in detecting rejection (27). We previously published a study examining five pediatric kidney transplant recipients monitored with dd-cfDNA over a two-year period. Our findings confirmed that dd-cfDNA peaked earlier than serum creatinine in those undergoing subAR, indicating that dd-cfDNA is more sensitive to kidney injury (32). We determined that dd-cfDNA provides a less invasive method for early detection of subAR, reducing the need for surveillance (protocol) biopsies when dd-cfDNA levels are below the threshold of 1%. This approach allowed clinicians to carefully monitor pediatric patients without invasive biopsies, find subclinical rejection early when there is a significant relative change (>149%) and to formulate and initiate treatment and prevention plans (27, 32, 33). Overall, the outlined studies demonstrate the diagnostic value of dd-cfDNA in managing kidney transplant recipients.

Despite these findings, there is limited data examining the *prognostic value* of dd-cfDNA. The RADAR and ADMIRAL and studies showed that patients could be stratified into low or high-risk groups based on dd-cfDNA levels <0.5% or >0.5%, respectively (22, 26). The RADAR study showed patients with dd-cfDNA levels >0.5% paired with a borderline or TCMR 1A diagnosis were more likely to have recurrent rejection, develop dnDSA, and have worsening estimated glomerular filtration rate (eGFR) over time (26). In a similar fashion, analysis from ADMIRAL revealed that dd-cfDNA elevations >0.5% preceded dnDSA development a median of 90 days and that persistent elevations in dd-cfDNA was associated with clinically significant eGFR decline between 12–36 month (22). The study also demonstrated the value of longitudinal surveillance and relative change value rather than a statistical cutoff (22). While these data provided a means to stratify patients into low or high risk, they did not provide insight into long-term outcomes and overall allograft survival.

The primary aim of this study was to evaluate the association between donor-derived cell-free DNA levels, graft function and survival in kidney transplant recipients. Secondary exploratory analyses examined the relationships between dd-cfDNA levels and markers of alloimmune activity, including biopsy-proven rejection, DSA positivity, C4d deposition, and longitudinal eGFR trajectories. In this retrospective single center study, we stratified 257 patients based on their dd-cfDNA levels as a risk factor to correlate with rejection rates, long-term graft function and graft survival. Patients were stratified by their first (index) dd-cfDNA measurement into low (<0.50%, Green group), medium (≥0.5 to 0.99%, Yellow group), and high (≥1.0%, Red group) representing stable, medium risk and high-risk groups, respectively, regardless of whether the dd-cfDNA measurement was associated with a specific rejection episode. Our hypothesis was that a higher degree of injury to the organ would predict worse long-term function and allograft survival outcomes. Our results show that patients in the Yellow and Red groups had incremental increases in the frequency of biopsy proven rejection (BPAR; 125BPAR/197 total Biopsies). Moreover, the incidence of AMBR and C4d+ ABMR increased significantly from low to high-risk group. Our data also showed that the Red group had the worst predicted eGFR at the end of the study period. Finally, a similar trend emerged when examining overall predicted graft outcomes with the worst results predicted for the Red group. To our knowledge, this is the first study that shows patients with a single dd-cfDNA ≥1.0% have high rates of ABMR with CD4d deposition and overall worse predicted long-term consequences for graft function and survival. These data support the notion that longitudinal monitoring of patients with dd-cfDNA could lead to improved outcomes by identifying high risk patients for careful monitoring.

## Materials and methods

### Patient population

This 3-year cohort study was conducted between March 2019 and December 2021 on adult kidney transplant recipients with at least one dd-cfDNA measurement at the Erie County Medical Center (ECMC), Buffalo, NY. Age, gender, ethnicity, etiology of CKD, and transplant status are shown in **Table 1**. Laboratory testing data, including dd-cfDNA, serum creatinine, eGFR, DSAs, mean fluorescence intensity (MFIs), and human leukocyte antigens (HLAs), were retrieved using the center’s electronic medical records.

**Table 1:** Patient demographics.

Variable	Green (n=86)	Yellow (n=81)	Red (n=90)	P - value
Age in years (%)	53 (12.04)	53 (13.63)	50 (15.28)	NS
Gender				
Male (%)	59 (69)	43 (53)	52 (58)	NS
Female (%)	26 (31)	38 (47)	38 (42)	
Race				
White (%)	45 (53)	45 (56)	55 (61)	NS
AA (%)	35 (41)	29 (36)	20 (22)	< 0.05
Others (%)	5 (6)	7 (8)	15 (17)	NS
Etiology				
HTN (%)	19 (22)	24 (30)	17 (19)	NS
DM (I & II, %)	36 (42)	31 (38)	28 (31)	NS
FSGS (%)	7 (8)	3 (4)	14 (16)	< 0.05
PKD (%)	4 (5)	8 (10)	11 (12)	NS
IgA (%)	3 (3)	5 (6)	5 (6)	NS
Others (%)	17 (20)	10 (12)	15 (16)	NS
Transplant				
DDKT (LDKT)	74 (12)	72 (9)	79 (11)	NS
KP/KLP (>2)	5/0 (0)	5/0 (1)	3/1 (19)	<0.05
cPRA% (se)	8 (2.7)	22.5 (3.9)	28 (4.3)	P=0.0008
KDPI% (se)	53.2 (2.7)	51.5 (2.6)	47.8 (2.6)	NS
DSA (%)	19 (22)	15 (19)	27 (30)	NS

DDKT, deceased donor kidney transplant; LDKT, living donor kidney transplant; KP, kidney–pancreas transplant; KLP, kidney–liver–pancreas transplant.

One patient in the yellow group and one patient in the red group underwent re-transplantation. All patients in the green group received their first kidney transplant.

### Selection criteria for study cohort

**Figure 1:**
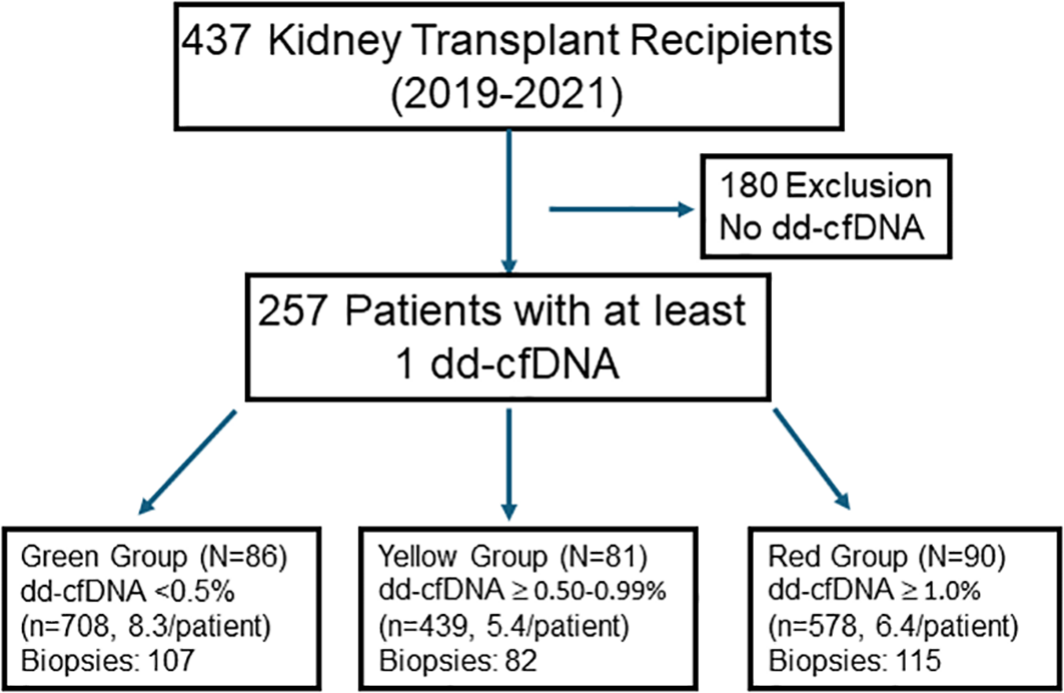
Schematic of patient inclusion and exclusion criteria for analysis. Data was examined for all patients followed between 2019 and 2021 with 257 patients having at least one dd-cfDNA test during that period. Patients were stratified into the Green, Yellow, and Red groups based on the indicated dd-cfDNA.

The study included kidney transplant recipients followed at ECMC between 2019 and 2021 (study window). To be included, patients needed to have at least 1 dd-cfDNA measurement during this time period (**Figure 1**). The selected cohort consisted of 257 adult patients with Chronic Kidney Disease (CKD, **Table 1**), a variety of genders, ethnicities, and etiologies of disease. These patients had received kidney transplants between 2004 and 2021 and had varying experiences with kidney biopsies from 2014 to 2022. Additionally, the study included patients who had experienced graft loss and returned to dialysis, as well as those who had passed away between 2019 and 2021. Patient demographics and chacteristics are outlined in **Table 1**, including gender, race, etiology of disease, transplant type, calculated panel reactive antibodies (cPRA), Kidney Donor Profile Index (KDPI) and DSA status at the time of tranpslant. cPRA and KDPI were determined using OPTN calculators.

Testing of dd-cfDNA was subject to each individual’s clinical needs based on surveillance and for-cause testing. To mitigate bias from heterogeneous testing times, we prespecified a cross-sectional analysis anchored at each patient’s index dd-cfDNA measurement within the study window. Patients were sub-grouped according to their first dd-cfDNA 1) If the test was <0.50%, patients were categorized in the green group, 2) if the test was ≥ 0.50-099%, patients were categorized in the yellow group, 3) if the test was ≥ 1.0%, they were categorized in the red group, representing stable patients, medium risk/subclinical rejection, and high risk for rejection, respectively.

### Measurements of dd-cfDNA

Venous blood was collected in Streck BCT cell free DNA Tubes at scheduled surveillance visits or for cause setting to determine the need for a kidney biopsy. Samples were shipped to the CLIA-certified laboratory at CareDx, Inc. (Brisbane, CA). Targeted next generation sequencing of pre-determined single nucleotide polymorphism was performed to quantify the donor fraction of cfDNA without requiring genotyping of donor or recipient (20, 21).

### Measurements of eGFR

eGFR was calculated using the 2021 CKD-EPI creatinine equation. Patients with at least three eGFR and over 3 years were included in the analysis. For eGFR, an average of monthly measurements was calculated and used for analyses.

### Renal allograft biopsies and classification of rejection

All biopsies were performed only when clinically indicated based on the treating nephrologist’s judgment. Biopsy triggers include unexplained rise in serum creatinine, new or worsening protinuria, concerning dd-cfDNA results (typically ≥1% or a rising trend), dnDSA, dd-cfDNA or a combination of these factors, symptoms suggestive of allograft dysfunction. For the purpose of this study, a biopsy was paired with a dd-cfDNA result if the dd-cfDNA samples was collected 30day or less before the biopsy. A total of 304 kidney transplant biopsies from 257 patients were examined. Biopsies with evidence of borderline changes or acute cellular rejection were selected for further analysis. The renal biopsy tissues for standard histology were fixed in Zamboni’s fixative and processed using automated tissue processors. Three-micron sections were cut and stained with hematoxylin and eosin, PAS, and trichrome stains. Biopsy reports were generated by the local nephropathologist based on the Banff 2019 classification criteria (34). The diagnoses of borderline changes, active T-cell-mediated rejection (TCMR), or antibody-mediated rejection (AMR) were based on the criteria defined in the 2019 Banff Working Group classification system (**Supplementary Tables 1**, **2**). For this study, the biopsies were re-examined and reported by three nephropathologists. Synoptic features were extracted from these reports for subsequent analyses. All kidney biopsies performed were summarized (**Supplementary Figure 1**). Histopathology examples for different categories of Banff Cellular and/or Humoral rejections were demonstrated (**Supplementary Figure 5****).** The study focused on evaluating rejection activity rather than chronicity. Therefore, tissue chronicity was not separately analyzed.

### Measurement of donor-specific antibodies, mean fluorescence intensity and human leukocyte antigens

The detection and quantification of DSAs were performed using a Luminex-based single antigen bead assay (KSL Biomedical). Luminex beads were sourced from One Lambda (Thermo Fisher Scientific, US), while the Luminex equipment was provided by FLEXMAP (Luminex Instruments, R&D Systems). Serum samples from patients were collected at various time points post-transplantation and incubated with beads coated with individual HLA antigens as per manufacturers instructions. These beads represent the specific HLA types of the donor. The beads were analyzed using a Luminex flow cytometer. DSAs with >1000 MFI were considered as a positive test.

### Statistical analysis

Descriptive statistics were calculated for each dd-cfDNA group. These included mean and standard deviation for age with associated Wilcoxon rank-sum tests, and group frequency counts for categorical measures with associated Chi-squared and Fisher’s exact tests (35, 36). Group comparisons were performed using Mann-Whitney analyses. Longitudinal mixed models were used to estimate the progression of dd-cfDNA and eGFR over time, while controlling for age, gender, race, and dd-cfDNA group (37). A logistic regression model for probability of graft failure was analyzed, controlling for age, gender, race, baseline eGFR, and dd-cfDNA (38).

## Results

### Study inclusion criteria and demographics

We performed a retrospective single center study examining data from 437 patients that were followed at our institution betweeen March 2019 and December 2021. Patients were eligible for inclusion if they had at least one dd-cfDNA measurement during the study period. Group assignment was based on each patient’s index dd-cfDNA value within the study window. All included patients were then monitored longitudinally for clinical outcomes (**Figure 1**). Patients were grouped according to their first dd-cfDNA levels as follows: Green (<0.5%), Yellow (0.5–0.99%), and Red (≥1.0%). These categories represent stable, low-risk, and high-risk status respectively, based on the range of dd-cfDNA test results described in the methods section (**Figure 1**).

The mean age of the patients was similar across the groups, with the Red group having an average age of 50±15.28 years, the Yellow group 53±13.63 years, and the Green group 53±12.04 years, with no significant difference observed between the groups (p>0.05) (**Table 1**). Gender distribution was also comparable among the groups with no significant difference found (**Table 1**, p>0.05). Racial composition showed a significant difference (p<0.05) in the percentage of African American (AA) patients (**Table 1**). The Red group had 61% White, 22% AA, and 17% other races. The Yellow group had 56% White, 36% AA, and 8% other races. The Green group had 53% White, 41% AA, and 6% other races (**Table 1**).

The primary end stage renal disease consisted of hypertension (HTN) and Diabetes mellitus (DM) most commonly, followed by Polycystic kidney disease (PKD) and IgA nephropathy (IgAN), with no significant differences (p>0.05) among 3 groups (**Table 1**). Focal segmental glomerulosclerosis (FSGS) was significantly different comprising 16% of the Red group, 4% of the Yellow group and, 8% of the Green group, (**Table 1**, p<0.05).

The distribution of transplant types and immunologic profiles among the Green, Yellow, and Red groups is shown in **Table 1**. The proportion of deceased donor kidney transplants (DDKT) and living donor kidney transplants (LDKT) was similar across all groups, with no statistically significant differences. Specifically, DDKT accounted for 86%, 89%, and 88% in the Green, Yellow, and Red groups, respectively, while LDKT comprised 14%, 11%, and 12%. Whereas most patients underwent kidney transplantation alone, 5 patients in the Green group, 5 patients in the Yellow group, and 3 patients in the Red group underwent combined kidney–pancreas transplantation. In addition, 1 patient in the Red group underwent combined kidney–liver–pancreas transplantation. With respect to transplant frequency, all patients in the Green group received a single transplant. One patient in the Yellow group received two transplants, whereas a total of 19 patients in the Red group received more than one transplant. Pre-Transplant cPRA levels were significantly higher in the Red group (28 ± 4.3) compared to the Green (8 ± 2.7) and Yellow (22 ± 3.9) groups (p = 0.0008), suggesting increased sensitization among Red group patients. Kidney Donor Profile Index scores and the presence of DSAs did not differ significantly across groups. These findings emphasize that, although transplant type and donor characteristics were similar among groups, the Red group had significantly higher pre-transplant immunologic risk as reflected by cPRA levels.

### Incremental increases in dd-cfDNA are associated with higher presence, activity, and severity of rejection

**Figure 2:**
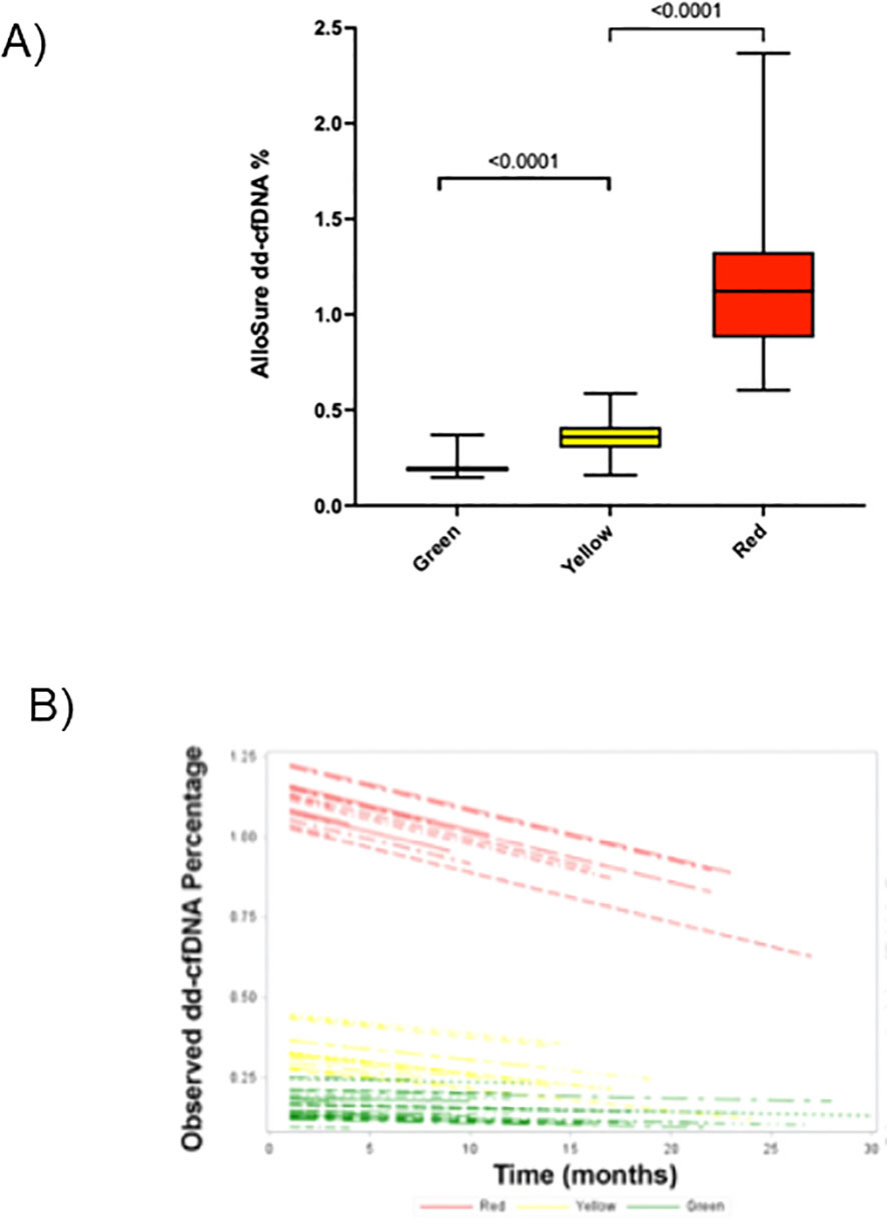
dd-cfDNA longitudinal data by patient group. **(A)** Combined monthly mean±SEM dd-cfDNA levels across study duration for each group. **(B)** dd-cfDNA levels are shown from the start of the duration of study period for each patient by group (Each line represents a single patient).

dd-cfDNA measurement is quantitative and indicative of the degree of cellular turnover or death that is actively occurring in the donor organ (21, 27, 39). We analyzed overall trends of monthly dd-cfDNA (mean±SEM) of each groups spanning from March 2019 through December 2021. The Green group had 708 dd-cfDNA measurements (8.3 tests/patient) and had fairly stable low dd-cfDNA levels of 0.19%±0.006% throughout the study period, while the Yellow group had 439 dd-cfDNA measurements (5.4 tests/patient) with levels of 0.40%±0.05% and the Red group had 578 dd-measurements (6.4 tests/patient) with the highest levels throughout the study with a mean of 1.2%±0.08% (**Figure 2A**). Examination of longitudinal mean dd-cfDNA levels of both the Red (1.85% to 1.25%) and Yellow (0.80% to 0.41%) groups showed a steady decline over time where as the Green (0.23% to 0.19%) group remained fairly stable for the duration of the study (**Figure 2B**).

**Figure 3:**
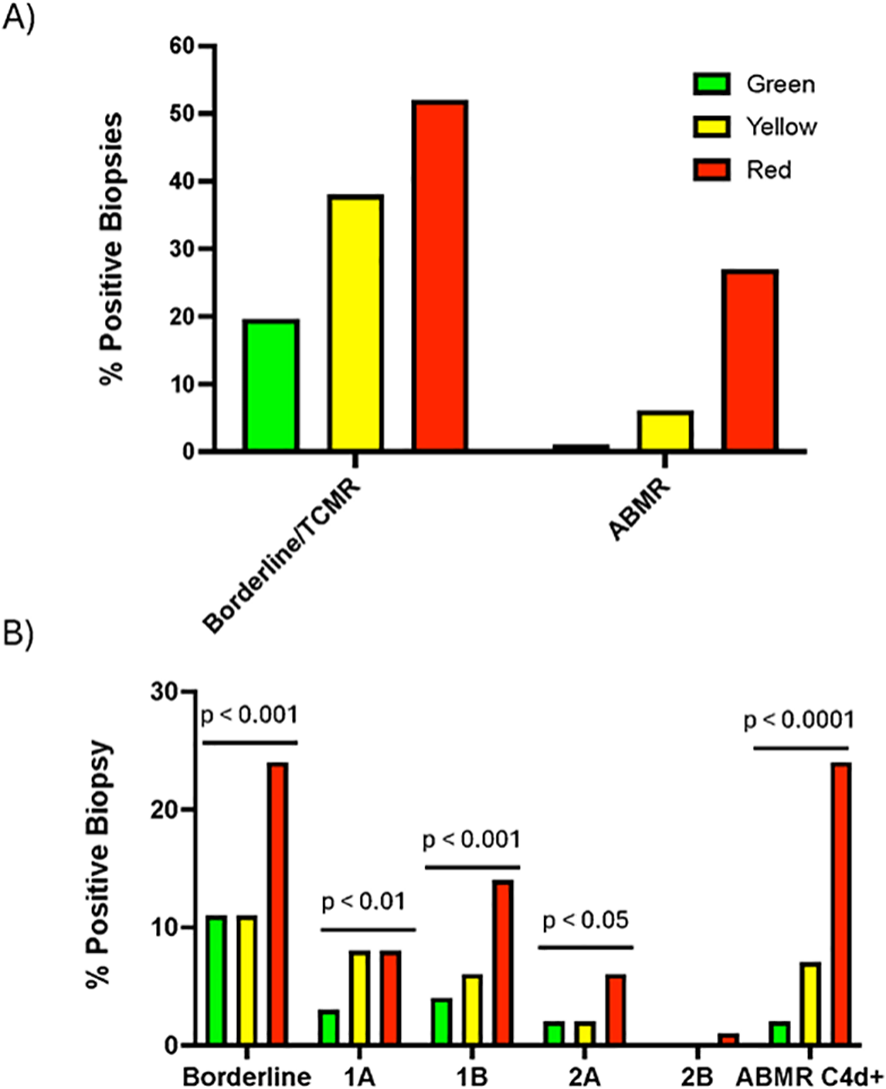
dd-cfDNA and biopsy proven rejection rates by group. The pathology reports for all biopsies by group were examined and scored based on the Banff 2019 criteria. **(A)** Biopsy frequency by broad classification and C4d+ are shown. **(B)** Biopsy frequency by specific classific.

Next, we examined the rate of biopsies per patient for each group, all of which were performed due to a specific clinical indication. Among all biopsies, a dd-cfDNA paired within 30 days or less were available for 61%, 48% and 63% for the Green, Yellow, and Red groups, respectively. The Green group included 85 patients amongst whom 84 had at least one biopsy and 1 who had no biopsies with a total of 107 biopsies performed (1.2± 1.0/patient, **Figure 1**; **Supplementary Figure 1**). The Yellow group included 81 patients amongst whom 57 patients had at least one biopsy and 24 had none with a total of 82 biopsies performed during the study period (1.0± 1.0/patient, **Figure 1**). The Red group comprised 90 patients with 68 having at least one biopsy while 22 had none with total of 115 (1.3± 1.3/patient). Overall, there was no difference in the rate of biopsies/patient performed when comparing the three groups, p=0.21 (**Figure 1**). When we examined the rate of acute rejection per biopsy, we found that the Green group had a 21% rejection rate, of which Borderline/TCMR made up 20% (21/107 biopsies) and ABMR made up 1% (1/107) (**Figure 3A**). The rate of cellular rejection almost doubled in the Yellow group with 38% (31/82) biopsies revealing either Borderline/TCMR and ABMR increased 6 fold to 6.1% (5/82), with an overall 44% rejection rate (**Figures 3A, B**). Finally, the Red group had the worst rejection rates, 81%, with 54% (62/115) of biopsies graded Borderline/TCMR and 27% (31/115) revealing ABMR (**Figure 3**). The Red group clearly were at high risk for TCMR and ABMR rejection with an increase of 2.5 and 27 fold compared to the Green group and increase of 1.4 and 4.4 fold compared to the Yellow Group, respectively. These findings are similar to a recently published study from the KOAR registry, which shows that higher levels of dd-cfDNA are associated with a higher likelihood of rejection in both the surveillance and for-cause biopsy settings, while low dd-cfDNA levels are associated with a lower likelihood of rejection, reflective of the high negative predictive value of the test (40). Hence, higher dd-cfDNA levels are more likely to be associated with rejection episodes and more likely to reveal a higher rate of rejection/biopsy performed, reflected by the difference in biopsy yield between the three groups (**Figure 1**, **Supplementary Figure 1**).

**Figure 4:**
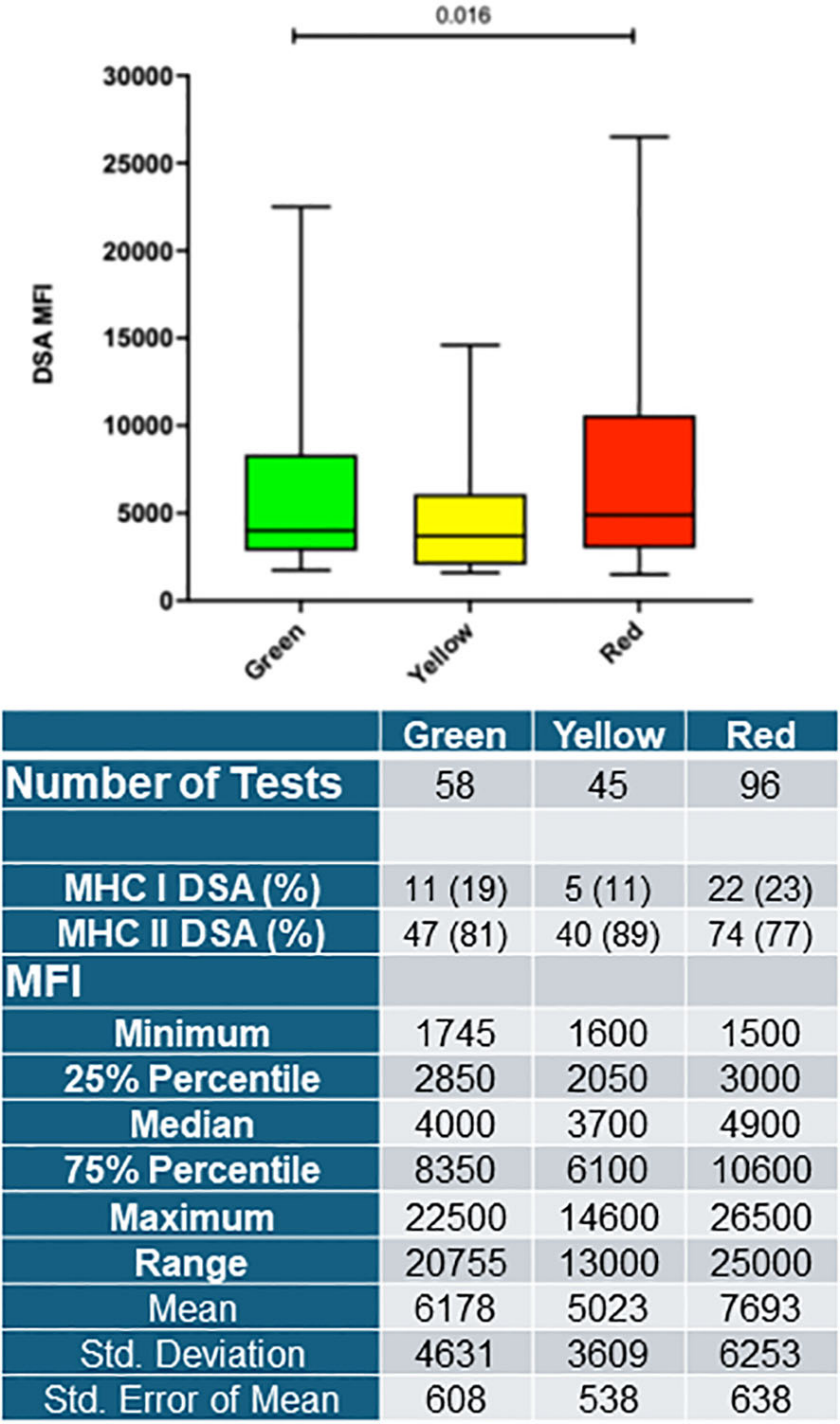
dd-cfDNA association with DSA detection and MFI. DSA positive tests were assessed for each group and classified into MHC I or MHC II. **(A)** Upper figure shows median and IQR MFI for each group for all DSAs detected. Lower table shows summary statistics for each group.

Given that there was a dramatic difference in rates of ABMR between the Red group compared to the Green and Yellow group, we closely examined the histological features of the biopsies. As expected, dd-cfDNA levels for specific types of rejection across the groups were significantly higher for each type of BPAR including Borderline/TCMR1A, TCMR1B>, and ABMR for the Red group (**Supplementary Figure 2A**). Amongs all biopsies, regardless if rejection was present, the median dd-cfDNA was also highest for the Red group, followed by the Yellow and Green groups (**Supplementary Figure 2B**). Intriguingly, we found a high frequency of C4d+ staining in the Red group biopsy tissue (18%) compared to the Yellow group (3.6%) and Green group (1.0%), (**Figure 3**). We also examined the frequency of all DSA reported for each group and the corresponding MFI for all patients available at the start of the study. DSA positivity was most frequent in the high dd-cfDNA (Red) group (**Table 2**). Examination of each group for the duration of the study revealed 22% (19/85 patients, 19% Class I, 81% Class II) had a DSA detected in the Green group, 19% (15/81, 11% Class I, 89% Class II) in the Yellow group, and 30% (27/89, 23% Class I, 77% Class II) in the Red group (**Figure 4**). The overal MFI of the detected DSAs for Green group was 4000 (IQR 2850-8350), Yellow group was 3700 (2050-6100), and Red group 4900(3000-10600) and was significantly different between the groups by analysis of variance, p=0.016 (**Figure 4**). We also performed a multi-variate analysis to determine whether patients grouped in Green, Yellow and Red groups were at higher risk of rejection. Our analysis showed the rejection odds ratio for Red was 1.6 and Yellow 1.3 compared to the Green group with only non-White race determined to have a higher odds ratio for rejection (**Supplementary Table 3**). These data demonstrate that stratifying patients based on incrementally higher dd-cfDNA ranges can identify those individuals at increasingly higher risk for both ACR and AMBR rejection rates, more severe histopathology, increased C4d deposition and higher rates of DSA positivity.

**Table 2:** Association between dd-cfDNA, DSA, C4D and HLA classes (I & II).

Variable	Green (n=86)	Yellow (n=81)	Red (n=90)	P-value
AMBR (+); [N (%)]				
Overall	8 (9.41)	4 (4.94)	14 (15.56)	0.0703
DSA (-)	8 (12.12)	1 (1.54)	6 (10)	0.0461
DSA (+)	0 (0)	3 (20)	8 (29.63)	0.0243
C4D (+); [N (%)]				
Overall	37 (43.53)	16 (19.75)	19 (21.11)	0.0008
DSA (-)	33 (64.71)	11 (16.92)	7 (11.67)	<0.0001
DSA (+)	4 (21.05)	5 (33.33)	12 (44.44)	0.2591
MFI; [Mean (SD)]				
C4D (+)	5804.17 (2804)	5864.8 (3029)	9040.63 (8181)	0.9674
C4D (-)	5234.78 (3033)	5216.83 (3768)	7468.17 (7324)	0.9602
HLA Class; [N (%)]				
Type I (A, B, C)	5 (26.3)	3 (20)	8 (29.6)	0.3706
Type II (DR, DQ, DP)	16 (84.2)	12 (80)	22 (81.5)	0.2786

### High levels of dd-cfDNA associate with declining eGFR and worse long-term graft survival

**Figure 5:**
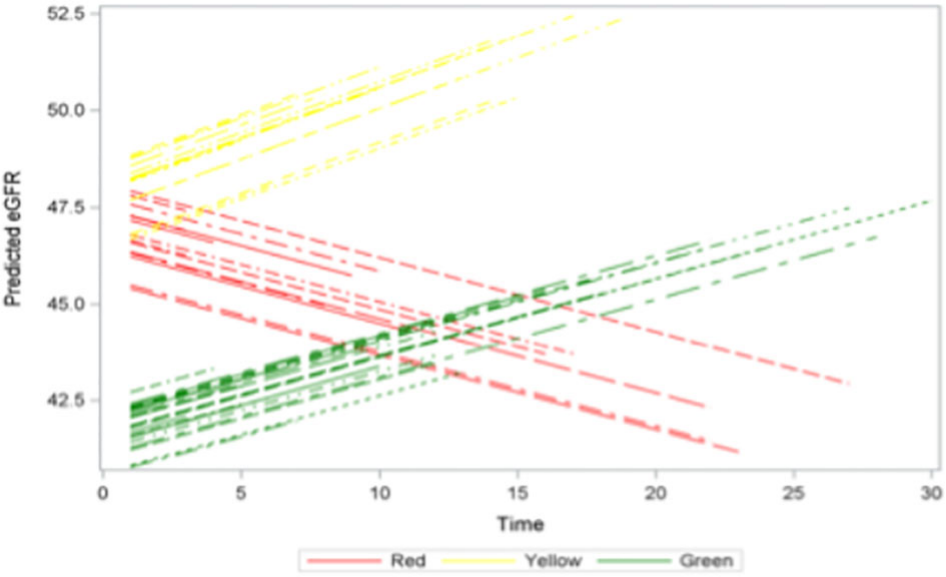
Predicted eGFR longitudinal data by patient group. Monthly eGFR levels for the study duration by group (Each line represents a single patient).

**Figure 6:**
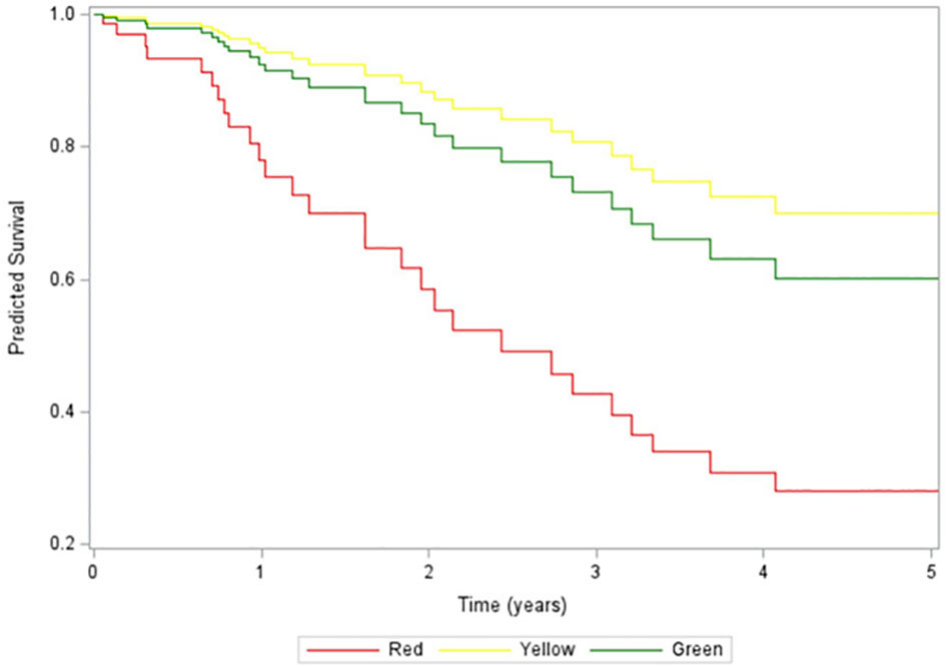
Death censored predicted graft survival data by patient group. Kaplan- Meir curve showing the predicted graft survival for each patient group over 5 years.

Several studies have previously associated higher dd-cfDNA levels with declines in allograft function over time (22, 26). However, there are no studies associating dd-cfDNA levels of patients with longer-term outcomes such as graft survival. Given our results above, which indicate that incrementally higher levels of dd-cfDNA are associated with higher rates and more severe rejection, we wanted to determine long-term graft function and survival for each of Green, Yellow and Red goups. We used patient data from each group to build a longitudinal mixed effects model predicting eGFR and logistic regression model to estimate the probability of graft failure. The model showed that the Red group had the worst predicted eGFR over time with a 6-point drop in eGFR over the study period (**Figure 5**). On the other hand, the Yellow group eGFR was found to improve in the same model with an 8-point increase in eGFR over the study period (**Figure 5**). Similarly, the eGFR for the Green group was estimated to have a 6-point increase in eGFR over the study period (**Figure 5**). Statistical analysiss did not show a significant difference in eGFR between the three groups (p=0.44). However, we did find a signficant difference if the Green and Yellow groups were combined and the analysis was restricted to white males, representing the majority of patients in the study, (**Supplementary Figure 3**). The Kaplan-Meir curve showing estimated graft survival was statistically significant between the three groups (p=0.0071) (**Figure 6****).** Next, we used a logistic regression model to estimate the probability of graft failure based on dd-cfDNA groups. We found that the Red group trended towards worse outcomes compared to the other two groups, however there was no significant effect of dd-cfDNA group on odds of graft failure (p = 0.5883), which could be limited due to the small number of deaths and graft loss found across the three groups (**Figure 6**). Only eGFR (p = 0.0426), were significantly associated with graft failure, with increasing eGFR associated with decreased rates of graft failure (**Figure 5**). However, when we combined the Yellow and Green groups and compared them to the Red group, we found a significant difference in the estimated probability of graft failure between the groups (p = 0.002) with eGFR significance improving further (p=0.033, **Supplementary Figure 4**). These results indicate that patients with dd-cfDNA >1% are more prone to have a decline in eGFR and and in turn could have worse graft survival over time compared to those patients with <1% dd-cfDNA levels.

## Discussion

Early detection of rejection in kidney transplantation is crucial for timely intervention to ensure the long-term survival of kidney allografts. However, conventional diagnostic methods, including measurement of serum creatinine, proteinuria and donor specific antibody are surrogate and unreliable markers of allograft rejection, and provide insufficient lead-time to prevent tissue damage, as they do not directly measure allograft injury (11, 13–15, 17, 18). Moreover, kidneys have significant reserve capacity and are very good at compensating for tissue loss without detectable functional decline (13, 14). Biopsies remain the gold standard to determine the status of the transplanted organ. However, biopsies are invasive, subject to heterogeneous tissue sampling, and prone to substantial inter-observer variability (9, 41, 42). Furthermore, there are no data showing a clear benefit for surveillance protocol biopsies, reflected by the fact that the majority of US transplant centers only perform them for clinical indication (9). Therefore, biomarkers that can directly detect allograft injury could provide a lead-time prior to substantial tissue injury and improve long-term outcomes.

Donor derived cell-free DNA is a biomarker which specifically detects allograft injury and rejection in transplant recipients (20). This diagnostic test offers advantages over conventional tests, as it is quantitative, precise, non-invasive, and has a short half-life (21). Numerous multicenter studies have obtained consistent performance of dd-cfDNA in detecting allograft rejection including a recent publication by Aubert et al. that included over 2800 patients with 3732 biopsies paired with dd-cfDNA testing (20, 26, 27, 39). This study also established for the first time that dd-cfDNA is an independent predictor of rejection, using a multi-variate analysis, with an odds ratio similar to patients with prior rejection, DSA, and better than creatinine and proteinuria (27). There is also limited data that examine the prognostic value of dd-cfDNA. The RADAR and ADMIRAL studies showed that dd-cfDNA levels >0.5% paired with a biopsy were predictive of recurrent rejection, dnDSA development and eGFR decline (22, 26).

Here we performed a retrospective analysis of kidney transplant recipients with at least one dd-cfDNA measurement to assess whether stratifying patients based on low-Green group (<0.5%), medium-Yellow group (0.5-1%), and high-Red group (≥1%) levels of dd-cfDNA could provide insight into overall association with graft status and future outcomes. We noticed a 2x increase in the rate of rejection per biopsy from the Green to Yellow group, 20% vs. 38%, and a further doubling from the Yellow group to the Red group, 38% vs. 81%. Given that the Green and Red groups had essentially the same biopsy rates when dd-cfDNA results were available, these findings suggest that (1) providers, at least initially, did not rely solely on dd-cfDNA and continued to place greater weight on standard clinical assessments, and (2) the Green group was not under-biopsied despite having lower dd-cfDNA levels compared with the Red group. This minimizes the likelihood that differential biopsy practices introduced statistical bias into our analyses (**Supplementary Figure 2B**). Higher levels of dd-cfDNA were correlated with incrementally higher rates of ABMR jumping 6-fold from Green to Yellow groups, and to 27-fold from Green to Red groups. In addition, the Red group also had the highest rate of C4d+ histological staining compared to the Green and Yellow groups, 18-fold and 5-fold, respectively. A newly published 56 center study, Kidney Outcomes AlloSure Registry, demonstrates similar findings confirming that patients with AlloSure levels ≥1% have higher rates of rejection present on surveillance and for-cause biopsies (40). Notably, this is the first analysis that associates dd-cfDNA levels with C4d deposition. Similarly, The Red group also had the highest frequency of patients with DSAs (30%) compared to the Green (22%) and Yellow (19%) groups. These data suggest stratifying patients based on their dd-cfDNA levels identifies the likelihood, activity, and severity of rejection and anti-donor antibody they may experience. This parallels the findings of Aubert et al. (2024) that incremental increases in dd-cfDNA correlate with higher degree of injury reflected in higher TCMR and ABMR lesion scores on biopsies (27). The ADMIRAL study elucidated that an elevated dd-cfDNA (0.5% or more) tripled the risk of detecting dnDSA a median of 90 days later. Even with statistical adjustment, a 1% increase in dd-cfDNA was associated with a 20% rise in dnDSA development (22). Similar correlations were made by Stites et al. where a larger proportion of the high dd-cfDNA (41%) group had higher *de novo* DSAs than the low dd-cfDNA group (3%) (26). Moreover, high levels of dd-cfDNA often coincide with high levels of DSAs, both indicating ongoing ABMR (20, 22, 24, 27, 28). This is supported by Bloom et al. where ABMR (including mixed ABMR and TCMR) lead to significantly elevated dd-cfDNA compared to non-ABMR (including TCMR-only) levels (20, 43). Therefore, these data demonstrate that incremental increases in dd-cfDNA levels correlate with higher incidence of DSA, increased C4d deposition and possibly higher rates of ABMR. Monitoring these markers together can provide valuable insights into the immune status of transplant recipients.

While graft and patient survival in the first-year post-transplant have improved significantly over the past two decades, there is a precipitous decline in graft survival beyond the first year without significant improvement during the same era (4). Predicting which kidney transplant recipients will be at risk for decreased graft function and survival in the long run could allow for optimized management and improved long-term outcomes. Early subclinical rejection correlates with worse graft function, shorter graft survival and lower patient survival rates (7). However, rejection is most likely not the sole determinant of graft function and survival with any type of injury contributing to progressive declining rates and eventual graft failure. The American Society of Transplantation has defined a failing kidney allograft as one characterized by “stable but low allograft function, declining function (when there is irreversible and progressive decline in kidney function with anticipated allograft survival of less than 1 year), and return to kidney replacement therapy” (44). Four broad areas are considered in the context of a declining functioning graft: prognosis and kidney failure trajectory; immunosuppression strategies; management of medical and psychological complications; and choice of kidney replacement therapy or supportive care following graft loss (44). Elevation in dd-cfDNA is agnostic of the cause of injury which would make it a direct reflection of graft cellular turnover/death that can be easily tracked in a longitudinal fashion. Therefore, rising or persistently elevated levels of dd-cfDNA could serve as a quantitative marker associated with graft function and survival regardless of whether it is associated with rejection (22, 23, 26, 27). In our study, an elevation in dd-cfDNA beyond 0.5% was associated with a much wider spread in estimated glomerular filtration rate (eGFR) with dd-cfDNA levels greater than 1%, -Red group- with the possibility of functional decline and lower graft survival over a 3-year period (**Figures 5**, **6**; **Supplementary Figures 3**, **4**). A similar decline in eGFR was previously demonstrated by Stites et al. (2020) following a rejection event (26). The broader spread in later eGFR values suggests that a significant proportion of the population might have experienced a subsequent decline in eGFR, possibly indicative of chronic injury requiring closer attention. In addition, the ADMIRAL study demonstrated that an elevation in dd-cfDNA (0.5% or more) was significantly correlated with clinical and subclinical allograft rejection. Persistently elevated dd-cfDNA predicted a 25% decline in the estimated glomerular filtration rate over three years (22). Hence, dd-cfDNA levels reflect the degree of graft injury, including subAR, which can precede changes in eGFR (27, 39). By correlating dd-cfDNA levels with eGFR measurements, we could establish a model that could be used longitudinally to identify and assess the possibility of declining function leading to improved outcomes and quality of life for transplant recipients.

In summary, stratification of patients using dd-cfDNA levels could help identify patients greatest at risk to devoloping rejection, possible decline in graft function and graft loss that would allow for modification of management strategies to improve outcomes. Longitudinal monitoring of kidney transplant recipients with a quantitative injury marker could be predictive of early declining function before it manifests in eGFR changes and graft loss. Larger multi-center studies are required in order to confirm our findings and solidify the direct relationship between increasing levels of dd-cfDNA and its prognostic value to predict declining eGFR and graft survival.

### Limitations

This study has several limitations. First, it is a retrospective, single-center observational cohort, and causal inference cannot be established. Patients were stratified into dd-cfDNA groups based on their first available measurement within the study window. Although this approach reflects real-world clinical workflow, we cannot discount prior immunological or acute injury episodes that could have impacted outcomes. In addition, the number of graft failure events was limited, constraining the statistical power of the multivariable model. Given this low event count, the resulting estimates should be interpreted as exploratory rather than confirmatory. Finally, as with all observational studies, residual confounding and unmeasured clinical factors may contribute to the associations observed.

Despite these limitations, the study provides novel real-world data on the association between dd-cfDNA and graft survival, as well as key markers of alloimmune risk in kidney transplant recipients.

## Data Availability

The raw data supporting the conclusions of this article will be made available by the authors, without undue reservation.
