# Supplementary figures and images for: Risk stratification by donor-derived cell-free DNA: a three-year single center study of kidney transplant outcomes from 257 patients
Source: Front Immunol. 2026 Feb 13;17:1737024. doi: 10.3389/fimmu.2026.1737024 (PMC12945818; doi:10.3389/fimmu.2026.1737024)

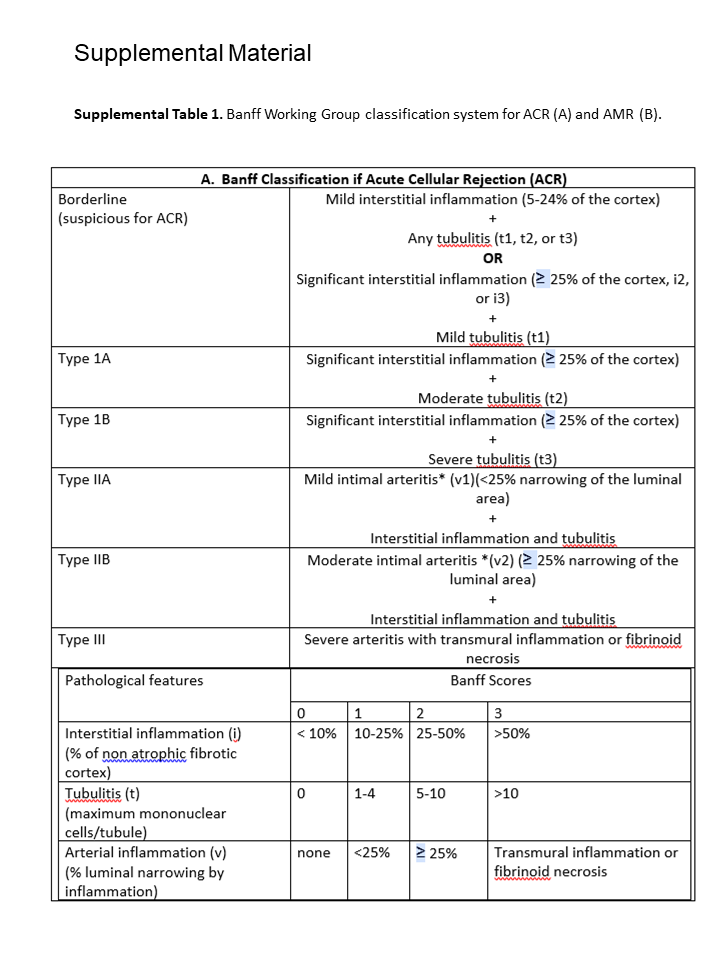

Supplement: Supplementary file 1 [file Image1.tif]

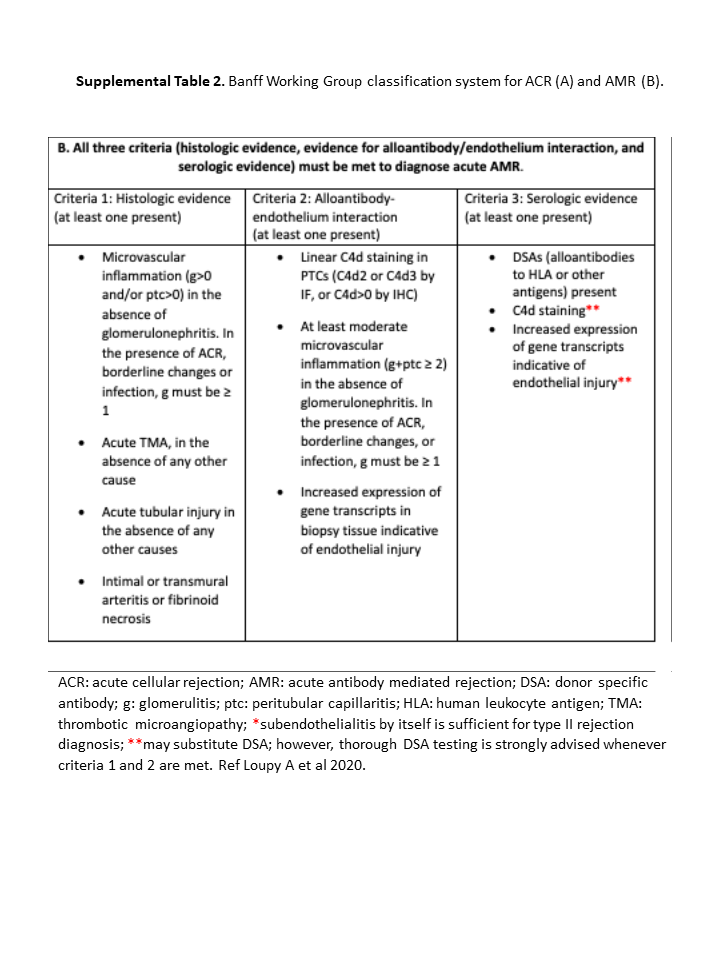

Supplement: Supplementary file 2 [file Image2.tif]

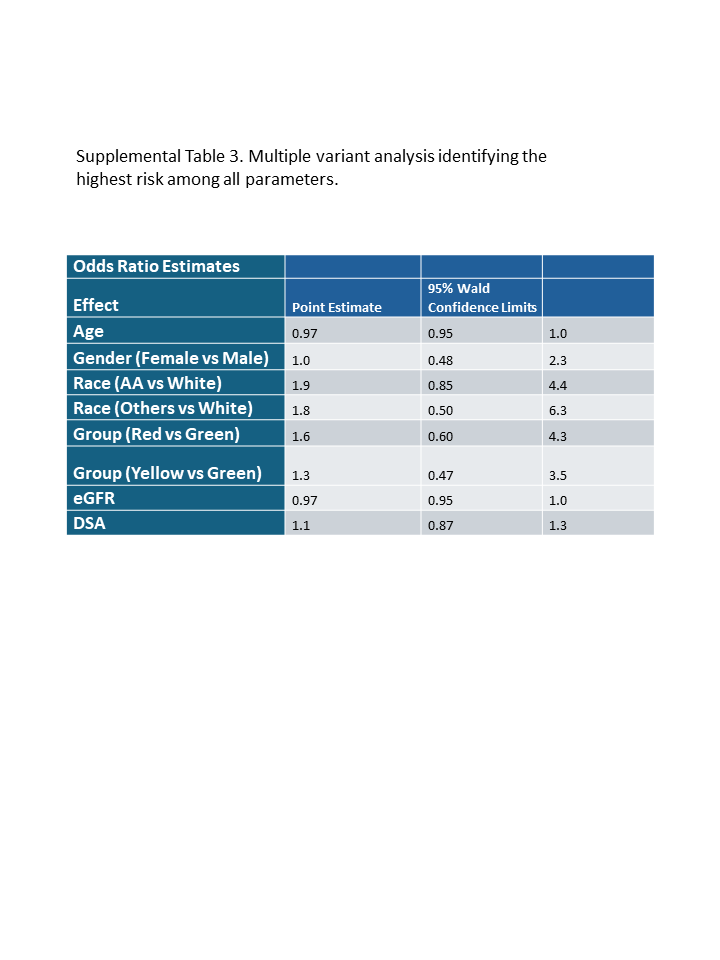

Supplement: Supplementary file 3 [file Image3.tif]

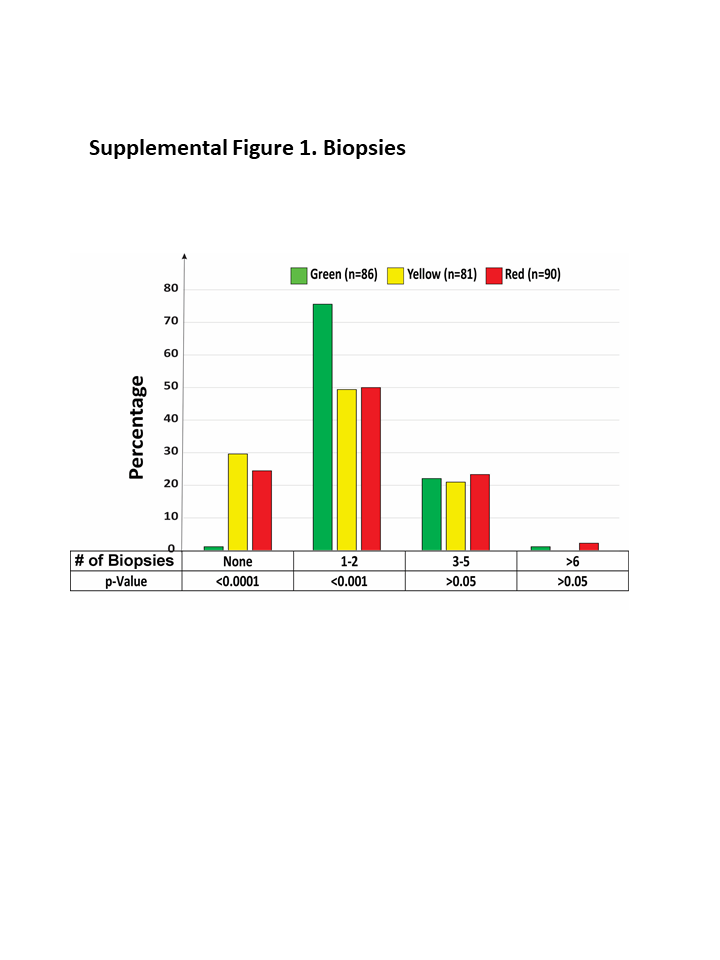

Supplement: Supplementary file 4 [file Image4.tif]

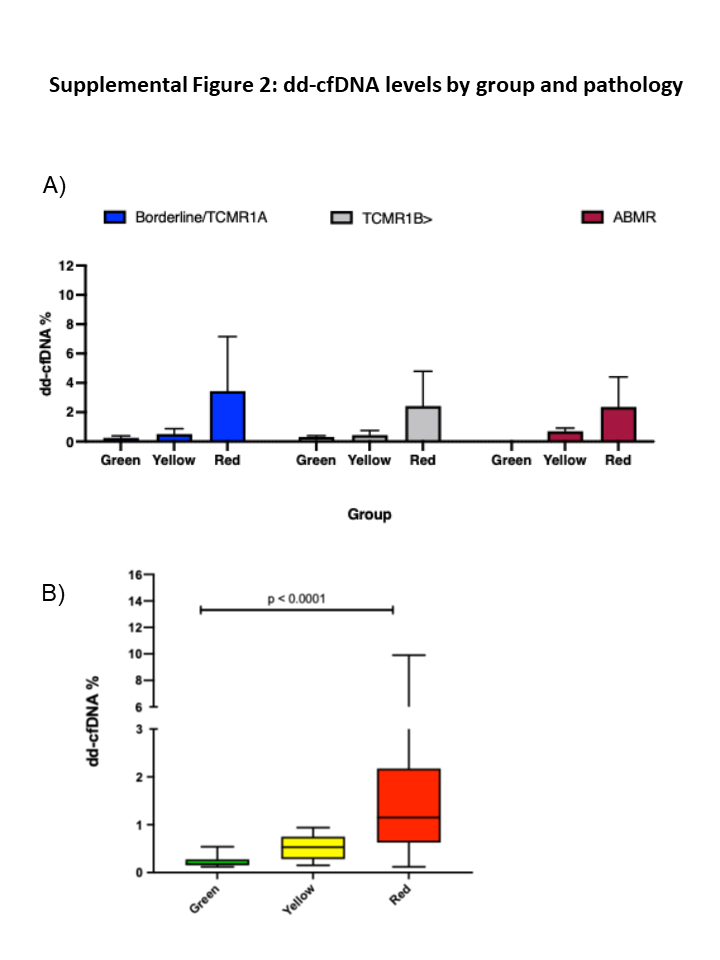

Supplement: Supplementary file 5 [file Image5.tif]

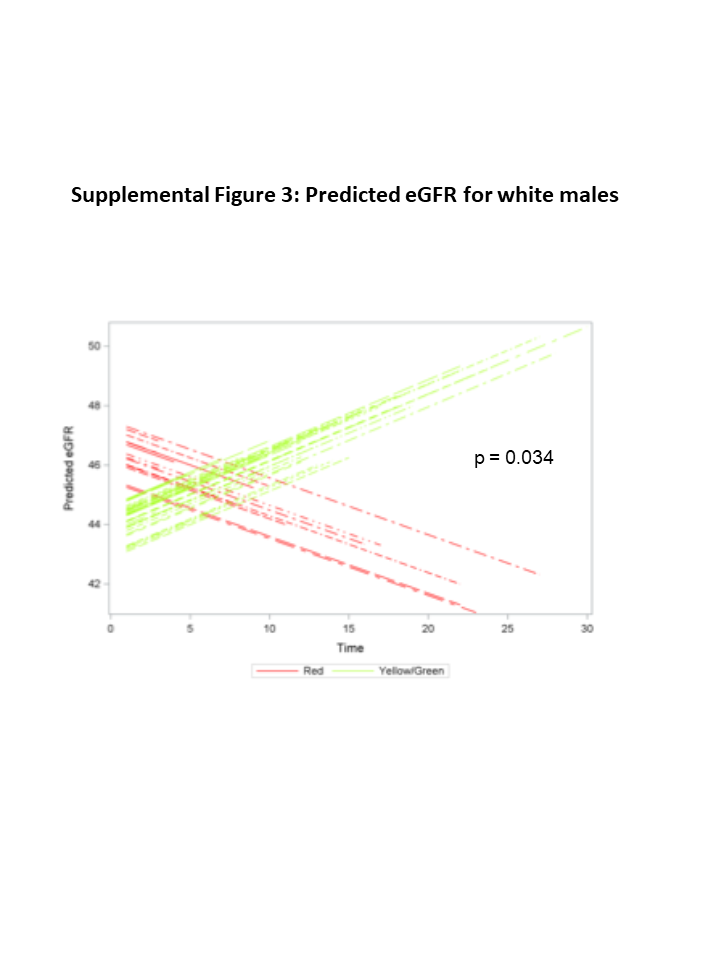

Supplement: Supplementary file 6 [file Image6.tif]

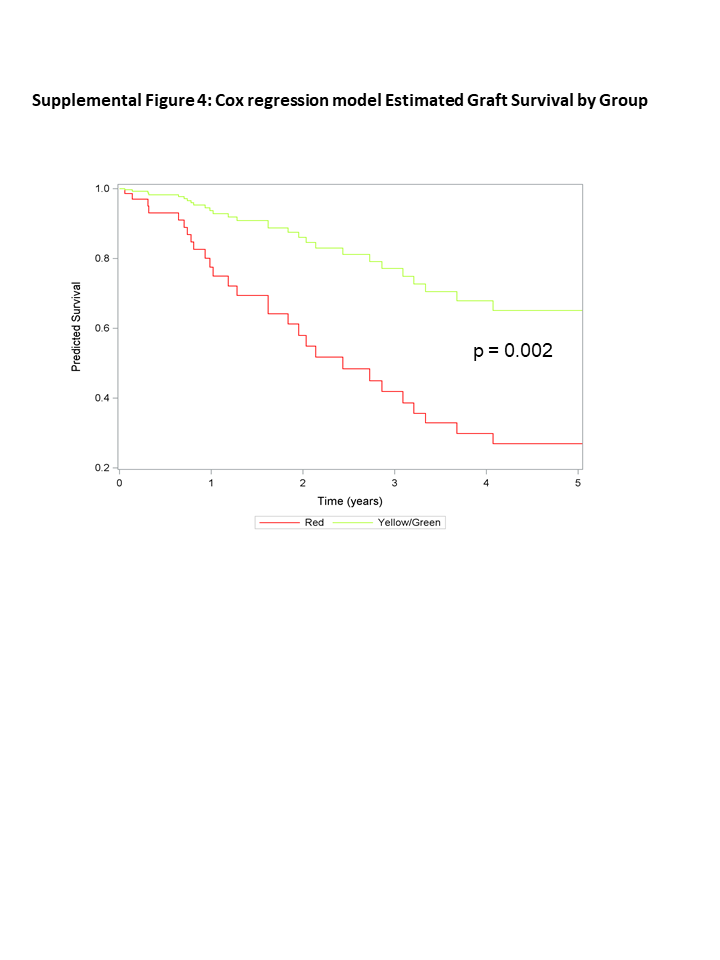

Supplement: Supplementary file 7 [file Image7.tif]

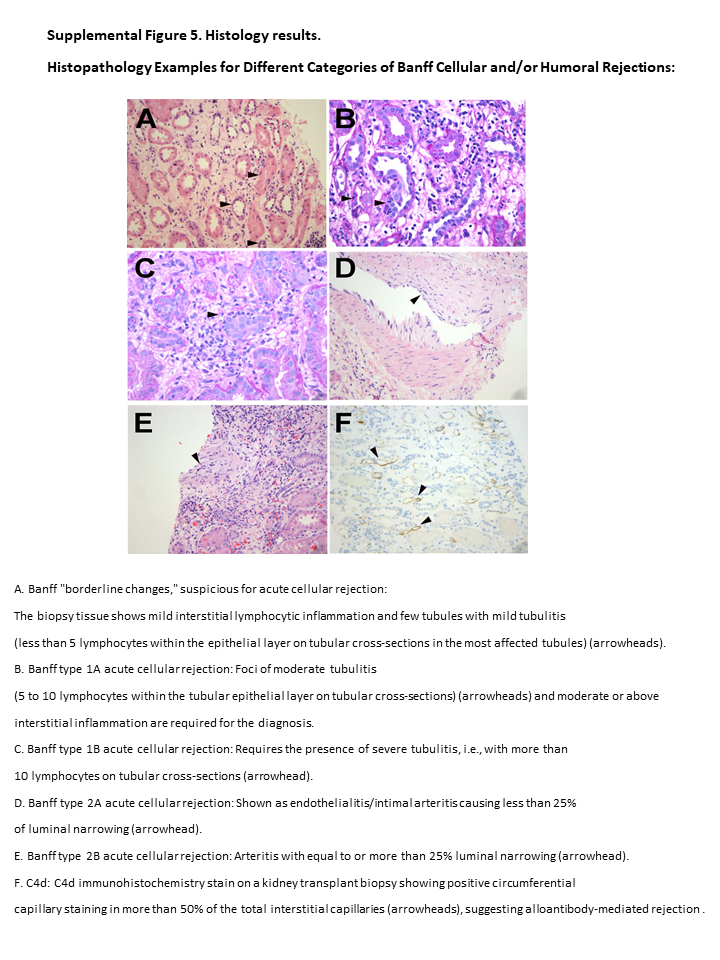

Supplement: Supplementary file 8 [file Image8.tif]
